# Photosynthetic traits of Australian wild rice (*Oryza australiensis*) confer tolerance to extreme daytime temperatures

**DOI:** 10.1007/s11103-021-01210-3

**Published:** 2022-01-08

**Authors:** Aaron L. Phillips, Andrew P. Scafaro, Brian J. Atwell

**Affiliations:** 1 Waite Research Institute and School of Agriculture, Food, and Wine, University of Adelaide, Adelaide, SA Australia; 2 ARC Centre of Excellence in Plant Energy Biology, Research School of Biology, The Australian National University, Canberra, ACT Australia; 3 Department of Biological Sciences, Macquarie University, Sydney, NSW Australia; 4 ARC Centre of Excellence in Plant Energy Biology, School of Agriculture, Food, and Wine, The University of Adelaide, Adelaide, SA Australia

**Keywords:** Photosynthesis, *Oryza sativa*, *Oryza australiensis*, Heat tolerance, Rubisco, Rubisco activase

## Abstract

**Key message:**

A wild relative of rice from the Australian savannah was compared with cultivated rice, revealing thermotolerance in growth and photosynthetic processes and a more robust carbon economy in extreme heat.

**Abstract:**

Above ~ 32 °C, impaired photosynthesis compromises the productivity of rice. We compared leaf tissues from heat-tolerant wild rice (*Oryza australiensis*) with temperate-adapted *O*. *sativa* after sustained exposure to heat, as well as diurnal heat shock. Leaf elongation and shoot biomass in *O*. *australiensis* were unimpaired at 45 °C, and soluble sugar concentrations trebled during 10 h of a 45 °C shock treatment. By contrast, 45 °C slowed growth strongly in *O. sativa*. Chloroplastic CO_2_ concentrations eliminated CO_2_ supply to chloroplasts as the basis of differential heat tolerance. This directed our attention to carboxylation and the abundance of the heat-sensitive chaperone Rubisco activase (Rca) in each species. Surprisingly, *O*. *australiensis* leaves at 45 °C had 50% *less* Rca per unit Rubisco, even though CO_2_ assimilation was *faster* than at 30 °C. By contrast, Rca per unit Rubisco doubled in *O. sativa* at 45 °C while CO_2_ assimilation was slower, reflecting its inferior Rca thermostability. Plants grown at 45 °C were simultaneously exposed to 700 ppm CO_2_ to enhance the CO_2_ supply to Rubisco. Growth at 45 °C responded to CO_2_ enrichment in *O. australiensis* but not *O. sativa*, reflecting more robust carboxylation capacity and thermal tolerance in the wild rice relative.

**Supplementary Information:**

The online version contains supplementary material available at 10.1007/s11103-021-01210-3.

## Introduction

Climate models predict increasing mean maximum temperatures globally, along with more frequent and intense heatwaves (IPCC 2019). Over the past century, the number of days of extreme heat in northern Australia has risen (CSIRO 2018). As average daily temperatures rise and spasmodic heatwaves become more frequent in agricultural systems, plant productivity declines (Dhir 2018), especially in cropping regions at low latitudes (e.g. India, Africa and Australia; Mahlstein et al. 2011; Jagadish et al. 2012). For example, wheat yield is expected to fall by 6% for every 1 °C increase in average daily temperature (Asseng et al. 2015), with losses of up to 10% per 1 °C possible in other crops (Dhir 2018). Similarly, in rice, grain yield declines by 10% for every 1 °C increase in mean temperature during the dry season (Peng et al. 2004). Heat stress therefore threatens food security (Tilman et al. 2011; Fischer et al. 2014). Despite this knowledge, there is limited research into the impact that long-term heat exposure has on plant physiology, with most studies focussing on transient exposure to high temperatures.

Heat stress affects whole-plant productivity through its impact on carbon metabolism. Extended periods of above-optimal temperatures suppress net carbon gain by impairing photosynthesis relative to dark respiration, reducing whole-plant growth and yield (Wahid et al. 2007; Campbell et al. 2007). Typically, respiration increases with rising temperatures, while photosynthesis declines because it is particularly sensitive to heat (Tjoelker 2018). For example, reduced growth of sugarcane at high temperature has been ascribed to lower rates of carbon assimilation and faster respiration (Ebrahim et al. 1998; Wahid et al. 2007; Gomathi et al. 2013). Similarly, the growth rates of domesticated rice (*Oryza sativa*) (Scafaro et al. 2016) and cotton (*Gossypium hirsutum*) (Reddy et al. 1992) leaves were reduced once air temperatures rose above about 35 °C. While respiration acclimates to changes in ambient temperature (Atkin and Tjoelker 2003), acclimation of photosynthesis to heat is less widely accepted (Chi et al. 2013; Benomar et al. 2018), although newly formed rice leaves (cv. IR64) appeared to acclimate to 40 °C days (Rashid et al. 2020). Nonetheless, the limits of thermotolerance may soon be reached, even in warm-climate species (e.g. rice), necessitating the identification of traits that can be used to increase tolerance to high temperature (Atwell et al. 2014; Fischer et al. 2014). A priori, the best candidate species are crop congeners collected from the hottest biomes. Hence, this study investigates an endemic Australian wild species of rice (*O*. *australiensis*) that has evolved in northern Australia. Scafaro et al. (2016) reported faster instantaneous leaf elongation rates linked to photosynthetic biochemistry during short bursts of heat shock in this species. The mechanistic basis of tolerance to *sustained* high daytime temperatures throughout vegetative development in this heat-tolerant species remains unknown and is addressed in this paper.

Plants adapted to savannahs and deserts have evolved a range of mechanisms to tolerate extreme heat, ranging from development and specialised leaf morphology and physiology (e.g. stomatal responses) (Wright et al. 2017) through to the accumulation of polymorphisms in heat-prone enzymes (e.g. for increased stability at high temperature) and changes in gene expression. This is well-documented in the case of heat shock proteins and their transcription factors (Scharf et al. 2012). Another mechanism for adaptation to heat (> 30 °C) is a more thermally stable Rubisco activase (Rca), a AAA^+^ protein that hydrolyses ATP while removing inhibitory sugar phosphates from the active sites of Rubisco, facilitating CO_2_ fixation (Portis 2003). Species that express high-temperature-stable isoforms of Rca might minimise impairment of photosynthesis during heat (Feller et al. 1998; Salvucci and Crafts-Brandner 2004; Scafaro et al. 2019). Regulation of in vivo Rca activity and abundance is also likely to be important, for example increasing the expression of endogenous Rubisco and Rca in tandem rescues heat-sensitive *O. sativa* from the deleterious effects of heat on photosynthesis and biomass accumulation (Qu et al. 2021). Despite this, the amount of endogenous Rca protein has not been explored as a pathway to photosynthetic thermotolerance in domestic and wild rice species adapted to a range of thermal regimes.

To understand the effects of extreme temperatures on carbon assimilation and utilisation, we raised atmospheric CO_2_ concentrations and measured growth and photosynthetic rates of heat-stressed (long- and short-term stress) *O. sativa* (domestic) and *O. australiensis* (wild) plants. We hypothesised that *O. australiensis* should withstand high daytime temperatures through a more robust photosynthetic metabolism. To assess the heat-resilience of photosynthesis, we used *A*:*C*_i_ curves, point measurements of assimilation rates and leaf elongation rates (LERs). Temperature effects on respiration, photorespiration and electron transport capacity were also characterised. Soluble sugar status of leaves and biomass accumulation in shoots and roots over 4 weeks are reported to establish the relationship between steady-state sugar levels and growth. Finally, we used quantitative proteomics to determine whether there were temperature-dependent changes in total Rca abundance between *O. sativa* and *O. australiensis*. This study presents empirical evidence in support of the superior heat tolerance in *O. australiensis*. We provide further support for photosynthesis being more robust in the wild species under heat and ascribe tolerance to a species-specific response of Rca expression. We also rule out other biochemical/biophysical processes such as respiration and electron transport rate (ETR) as factors that explain the thermotolerance of *O. australiensis*.

## Methods

### Plant material

*Oryza sativa* ssp. *japonica* cv. Amaroo and the wild tropical species *O. australiensis* (sourced from Keep River, in the northernmost region of Western Australia; 15° 58′ S, 129° 03′ E) were germinated at 36 °C overnight and sown in 1.65-L pots. The soil, a fine-textured krasnozem (sourced locally from Robertson, NSW, Australia), was kept moist at all times by keeping the pots in shallow trays of water. Plants were watered once a week with a commercial water-soluble fertiliser at a concentration of 1 g L^−1^ (AQUASOL, Yates, Australia). Seedling leaves were sprayed once with dilute, freshly prepared ferrous sulphate solution to prevent iron deficiency.

### Heat-shock experiments

In *heat-shock* experiments, plants (*n* = 3) were grown for 6 weeks in a glasshouse under a day/night temperature regime of 30/22 °C, with a midday photosynthetic photon flux density (PPFD) of 1106 ± 13 μmol quanta m^−2^ s^−1^ and a 12-h light period. Unless specified, all measurements and samples were taken over the course of 1 day. Plants were transferred to large growth chambers (model no. PG. 15. 18.9.TD + C [rt]0.3 × 1000.2 × 400.R; Thermoline Scientific Equipment, NSW, Australia) on the day of measurement. Two hours prior to the photoperiod, chambers were set to 30 °C. At the beginning of the photoperiod one cabinet was set to 45 °C (a second cabinet was maintained at 30 °C to act as a control). Unless specified otherwise, the relative humidity in all growth chambers was maintained between 60 and 70% during the photoperiod.

### Shoot and root dry biomass in heat-acclimated plants

Seeds of *O*. *sativa* and *O*. *australiensis* were germinated and maintained in glasshouse conditions as above for 1 week. After 7 days, *O. sativa* and *O. australiensis* seedlings (*n* = 20) were harvested for baseline measurements of shoot and root fresh and dry mass. Seedlings were exposed to 25, 35 or 45 °C for 4 weeks in growth chambers and harvested weekly (*n* = 6).

To explore the effect of CO_2_ fertilisation on *O. sativa* and *O. australiensis*, 6-week-old plants (*n*_30 °C_ = 16; *n*_45 °C_ = 4) were maintained in 700 ppm CO_2_, and 30 or 45 °C during the light period. Shoot and root dry mass were determined when the plants were 12 weeks old. CO_2_ levels were set and maintained using a custom-made CO_2_ solenoid (The Canary Company, Lane Cove, Australia).

### Leaf elongation rates: short- and long-term response to heat

Seedling growth was tracked through measurements of LER using a HR4000 Linear Variable Displacement Transducer (LVDT) with data logged every 3 min by the program VuGrowth ver. 1.0 (Applied Measurement, Oakleigh, Vic). Three experiments were conducted for the measurement of LERs.

In the first experiment (heat shock), 6-week-old plants (*n* = 3) at the four- to five-leaf stage were moved to a growth cabinet (containing the LVDT apparatus) set to the conditions described above. The youngest, fully expanded leaf of each plant was measured for 12 h and the same six plants (three per species) were used throughout the measurement period. Heat shock (45 °C) was imposed as described above. Leaf growth was measured for 8 h at 45 °C, and for 2 h at 30 °C. LERs were calculated using increase in leaf lengths for each 1-h period. At 1, 5 and 10 h into the photoperiod leaves from these plants were used for point measurements of photosynthesis, sugar and Rca analysis.

In the second experiment, plants (*n* = 5–8) grown at 25, 35, or 45 °C for 4 weeks were moved sequentially into the LVDT chamber. Measurement of LER commenced 1 h into the photoperiod. Leaf elongation was measured for 8 h (8:00–16:00) and LERs were calculated as the rate of elongation over that time.

In the third experiment, the CO_2_ level in the LVDT chamber was increased to 700 ppm. Plants (*n* = 4) of *O. sativa* and *O. australiensis*, which had been grown at 30 or 45 °C and 700 ppm CO_2_ for 6 weeks, were moved (independently) to the high-CO_2_ LVDT chamber for LER measurements and measured under their respective growth conditions. Measurements were made as above and were replicated three times (total *n* = 12).

### Sugar extraction and determination

Leaves of *O. sativa* and *O. australiensis* grown at 30 °C or exposed to 45 °C as a shock treatment were used for sugar analysis (see above). One fully expanded, healthy leaf (~ 200 mg) was excised from plants at each time point (1, 5 and 10 h into the light period; *n* = 3), snap-frozen in liquid nitrogen and stored at − 80 °C. Soluble sugar content was determined following the anthrone method (Yemm and Willis 1954) using glucose standards (25, 50, 75 and 100 mg glucose into 1000 mL H_2_O).

### Gas exchange: rapid A:Ci response (RACiR) curves, Laisk curves and point measurements

*Oryza sativa* and *O. australiensis* used for the generation of RACiR curves were grown in glasshouses (as described above) for 7 weeks. Plants (*n* = 4–6) were acclimated to the desired conditions in growth cabinets (Model BDR16; Conviron, Manitoba, Canada) set to 30 or 45 °C and 400 ppm or 700 ppm CO_2_ for 2 weeks prior to measurement (plants were 9 weeks old when measured). Plants grown at 30 °C were exposed to 45 °C following their measurement in the steady state to form the heat shock group.

RACiR curves were made following the procedure of Stinziano et al. (2017) and Stinziano et al. (2019) on a LI-6800 (Li-Cor, Lincoln, USA) with modifications. Briefly, to measure CO_2_ assimilation (*A*_*n*_), conditions in the IRGA head were set to match the conditions of the growth chamber (block temperature of 30 or 45 °C; reference CO_2_ level of 400 or 700 ppm), with a flow rate of 500 μmol air s^−1^, relative humidity of ~ 70%, a fan speed of 10,000 rpm, with a photosynthetic photon flux density (PPFD) of 1500 μmol quanta m^−2^ s^−1^. For curve generation, reference CO_2_ was reduced to 10 μmol mol^−1^ air and increased to 1010 μmol mol^−1^ air over a 10-min period (ramping = 100 μmol CO_2_ mol^−1^ air min^−1^). Measurements were taken by the LI-6800 every two seconds. All RACiR curves were corrected with empty chamber measurements. Leaves used for RACiR measurements were also used for Laisk curves and fluorescence measurements.

RACiR curves were initially converted from intercellular CO_2_ partial pressure (*C*_i_) to chloroplastic CO_2_ partial pressure (*C*_c_) using mesophyll conductance (*g*_m_) and its temperature response for *O. sativa* and *O. australiensis* as reported by Scafaro et al. (2016). The subsequent *A*-*C*_c_ (where *A* is the CO_2_ assimilation rate measured by the LI-6800) curves were analysed using the standard C_3_ photosynthesis model (Farquhar et al. 1980),

$$A_{c} = V_{{{\text{cm}}ax}} \times \frac{{C_{c} - \varGamma ^{*} }}{{C_{c} + K_{{{\text{air}}}} }} - R_{{{\text{light}}}}$$

$$A_{{\text{j}}} = J_{\max } \times \frac{{C_{c} - \varGamma^{*} }}{{4C_{c} + 8\varGamma^{*} }} - R_{{{\text{light}}}}$$

where *A*_c_ (carboxylation-limited CO_2_ assimilation rate) was fit to *C*_c_ partial pressures in the linear phase (corresponding to a mean *C*_c_ value of 38 Pa) and *A*_j_ (electron-transport limited CO_2_ assimilation rate) fit to *C*_c_ partial pressures above this point. *V*_cmax_ (maximum rate of carboxylation) and *J*_max_ (maximum rate of electron transport) were iteratively fit using a non-linear least-squares fit (R statistical). The Michaelis–Menten coefficient of Rubisco in air (*K*_air_) and its temperature response (value at 25 °C = 30.5 kPa; activation energy = 60.5 kJ mol^−1^) came from previous in vitro measurements in *O. sativa* (Hermida-Carrera et al. 2016). We assumed no difference in Rubisco kinetics between the two species. Respiration in the light (*R*_light_) and the CO_2_ compensation point in the absence of mitochondrial respiration (Γ*) were those measured by the Laisk method (Laisk 1977) (see below).

Laisk curves (*n* = 5–6) were generated with a LI-6800 (Li-Cor, Lincoln, USA). The curves were made with a *C*_i_ range of 50, 75, 100 and 120 ppm at three different light levels (PPFD = 100, 200 and 400 μmol quanta m^−2^ s^−1^). The intersection of the three lines was used to estimate *R*_light_ and Γ*.

Further, point measurements of gas exchange were made on *O. sativa* and *O. australiensis* using a LI-6400 (Li-Cor, Lincoln, USA) at saturating light levels (1500 μmol quanta m^−2^ s^−1^). All measurements were made 1, 5 and 10 h into the photoperiod on plants (*n* = 3) acclimated to 30 °C growth chambers (see above). Heat shock was imposed as described above. The CO_2_ in the reference chamber was set to 400 μmol CO_2_ mol^−1^ air and the block temperature was set to 30 or 45 °C.

### Chlorophyll fluorescence: dark and light adapted leaves

Following the RACiR and Laisk curves, the same leaves (*n* = 5–6) were used for determining chlorophyll fluorescence parameters as per instructions (see *Licor Support guidelines*; accessed September 2018). Leaves of interest were wrapped in aluminium foil and the lights in the growth cabinets were switched off. The plants were kept in the dark for 15 h (5:00 pm–8:00 am) before dark-adapted fluorescence measurements were made. Foil was removed from each leaf in the dark and the leaf was inserted into the chamber of a LI-6800 fitted with a fluorescence head. Minimal fluorescence (*F*_o_) and maximal fluorescence (*F*_m_) were measured with a rectangular flash of actinic light. Variable fluorescence (*F*_v_; estimated from *F*_*o*_ and *F*_m_) was used to calculate the maximal efficiency of photosystem II (PSII; *F*_v_/*F*_m_).

Dark-adapted leaves were then exposed to 1500 μmol quanta m^−2^ s^−1^ for 1 h to induce light acclimation. A Multiphase Flash™ (MPF) of actinic light was used in combination with a ‘dark pulse’ flash (i.e. a pulse of infrared light) to estimate *F*_s_ and *F*_m_’. MPF is a method for more accurately determining *F*_m_’ in light-adapted leaves, while the dark pulse allows for the calculation of *F*_o_’ (the minimal fluorescence in the dark of a light-adapted leaf; Loriaux et al 2013; Avenson and Saathoff 2018). These parameters were used to calculate the photochemical yield of PSII (*Φ*_PSII_) and the efficiency of PSII energy harvesting in the ‘open’ (oxidised) state (*F*_v_’/*F*_m_’). ETRs were derived from these values by the LI-6800.

### Quantifying relative abundance of RbcL and Rca by tandem mass spectrometry (MS/MS)

*Oryza sativa* and *O. australiensis* leaves (~ 200 mg; *n* = 3) sampled 1, 5 and 10 h into the photoperiod (see above) were ground to a fine powder in liquid nitrogen. Leaf powder was washed in ice-cold 10% TCA/0.07% DTT in acetone and incubated at -20 °C for 1 h. Following centrifugation at 4000×*g* for 45 min, supernatant was discarded and the pellet was washed with 0.07% DTT in acetone and stored at – 20 °C overnight. Samples were centrifuged at 4000×*g* for 45 min at 5 °C and the supernatant was discarded. Following resuspension in 0.07% DTT in acetone, samples were incubated at − 20 °C for 1 h. These washes were repeated twice. Pellets were air-dried, suspended in extraction buffer (250 mM TEAB/1.5% SDS), and stored at − 20 °C overnight. Pellets (kept on ice) were homogenised six times with a Precellys24 tissue homogeniser (Bertin Instruments, Montigny-le-Bretonneux, France) set to 6500 rpm for 20 s. Homogenised samples were centrifuged at 4000×*g* for 1 h at 10 °C. Total protein concentration of the supernatant was determined using a Direct Detect infra-red spectrometer (Merck Millipore, Darmstadt, Germany).

100 µL of 250 mM TEAB was added to 20 µg of sample. DTT was added to a final concentration of 10 mM. This mixture was incubated at 60 °C for 1 h. IAA was added to a final concentration of 20 mM, and samples were incubated at room temperature for 1 h in the dark. Trypsin (0.1 µg µL^−1^) was added in a 1:50 (trypsin:sample) ratio and incubated overnight at 37 °C. Digested samples were dried in a SpeediVac and resuspended in 250 mM TEAB. Pierce Detergent Removal spin columns (Thermo Scientific, IL, USA) were used to remove residual SDS. Samples were dried again in a SpeediVac and stored at − 20 °C. Prior to MS/MS analysis, samples were resuspended in loading buffer (2% acetonitrile (ACN); 0.1% formic acid).

Each sample (10 µL containing 3 μg of peptides) was injected onto a peptide trap (Bruker peptide Captrap) for pre-concentration and desalted with 0.1% formic acid, 2% ACN, at 10 μL min^−1^ for 5 min. The peptide trap was then switched into line with the analytical column. Peptides were eluted from the column using linear solvent gradients, with steps, from mobile phase A (0.1% formic acid): mobile phase B (99.9% ACN/0.1% formic acid) (98:2) to (90:10) for 10 min, then to (65:35), at 600 nL min^−1^ over a 78-min period. After peptide elution, the column was cleaned with 95% buffer B for 10 min and then equilibrated with 98% buffer A for 20 min before the next sample injection. The reverse phase nanoLC eluent was subject to positive ion nanoflow electrospray analysis in an Information Dependent Acquisition (IDA) mode.

In the IDA mode, a TOF–MS survey scan was acquired (m/z 350–1500, 0.25 s), with the ten most intense multiply charged ions (2 +–5 + ; counts > 150) in the survey scan sequentially subjected to MS/MS analysis. MS/MS spectra were accumulated for 50 ms in the mass range m/z 100–1500 with rolling collision energy.

### Data analysis

Data analysis was done in R (R Core Team 2017). All data, unless specified otherwise, were analysed by two- or three-way ANOVAs and Tukey HSD or Duncan’s Multiple Range tests. Figures were produced using R and GraphPad Prism (version 9).

The data collection and processing for mass spectrometry was conducted by the Australian Proteomics Analysis Facility (APAF; Macquarie University, Sydney, Australia). The LC–MS/MS data of the IDA runs of each sample were searched using ProteinPilot (v4.2; AB Sciex) in ‘thorough’ mode. MGF files (peak list) were exported, submitted to Mascot (Matrix Science, UK) and searched against NCBInr *O. sativa* (rice) database for Rca. Relative abundances for Rca and the large subunit of Rubisco (RbcL) were determined across samples using emPAI values, which were normalised against emPAI values of the total protein pools in *O. sativa* and *O. australiensis* (see Ishihama et al. (2005) and Zhu et al. (2010) for details on the emPAI method).

## Results

### Sequential destructive harvests

**Fig. 1:**
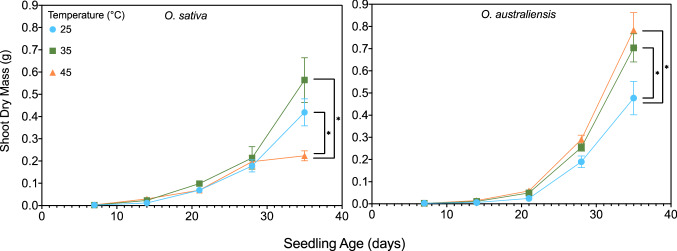
Shoot dry mass of *O. sativa* and *O. australiensis* plants grown at 25, 35, and 45 °C from 1 week of age for 4 weeks (35 days). Values are means ± SE; n_age=7_ = 20; n_age=14–35_ = 6. Asterisks denote final-harvest means that are significantly different within each species as determined by Tukey HSD

There was a significant three-way interaction between harvest time, temperature and species in shoot dry weight (*p* < 0.0001; Fig. 1). Follow-up two-way ANOVAs for each harvest time revealed that shoot dry weights of the two species first diverged after 35 d of exposure to 45 °C (*p* < 0.0001). *Oryza sativa* had significantly reduced shoot growth at 45 °C compared with plants growing at 25 and 35 °C. By contrast, *O. australiensis* shoots were significantly lighter at 25 than at 35 and 45 °C. Similar trends were seen in the root dry weight (Fig. S1), demonstrating slowest growth of *O. australiensis* was consistently at 25 °C whereas in *O. sativa*, plants grew slowest at 45 °C. The impact of the 45 °C treatment on the growth of *O. sativa* shoots became severe after 28 days. Similarly, differences in root mass were only detected in both species following 21 days exposure to treatment conditions.

### Leaf elongation rates: short term vs. long term response to temperature

**Fig. 2:**
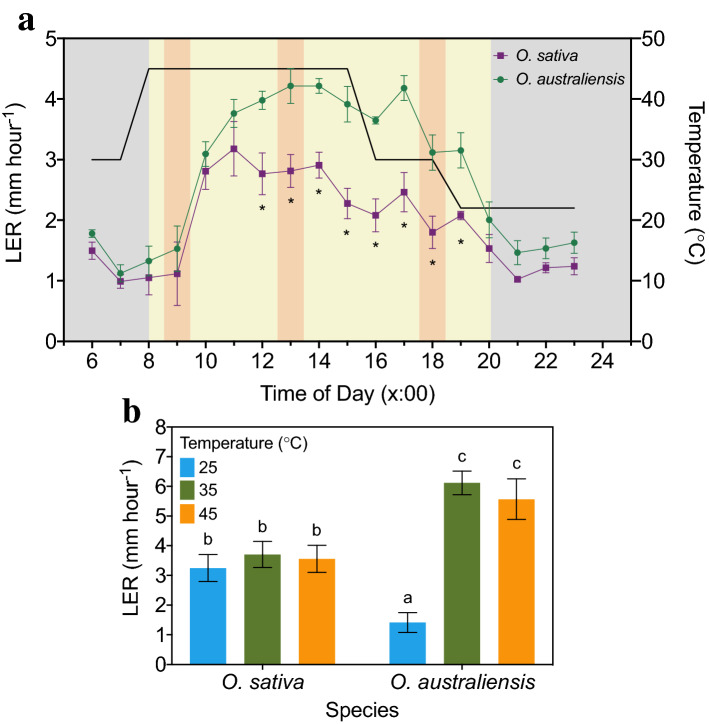
Effect of different growth temperatures on the elongation rates of leaves of wild and domestic rice. **A** Mean (*n* = 3; ± SE) leaf elongation rates (LERs) of *O. sativa* and *O. australiensis* plants exposed to 45 °C for 8 h (heat ‘shock’). Plants were grown at 30 °C daytime temperature for 6 weeks prior to the shock event. The same plants were measured across the sampling period. The solid black line shows the temperature of the growth cabinet. Grey and yellow shading indicates the photoperiod (lights off and on, respectively). Red shading indicates sample times (9:00, 13:00 and 18:00) for tissue collection for sugar and Rca analyses. Asterisks are used to indicate when *O. sativa* and *O. australiensis* LERs are significantly different. **B** Mean (± SE) daily LERs of *O. sativa* and *O. australiensis* plants grown at 25, 35 or 45 °C for 5 weeks (*n* for each group is reported in Table 1). In a second experiment, plants were measured in morning (8:00–12:00) and afternoon (12:00–16:00) groups to account for time-of-day effects. LERs were pooled across morning and afternoon groups because no difference was detected between the groups. Means that do not share a letter are significantly different (Duncan’s Multiple Range test)

A two-way repeated-measures ANOVA was conducted to determine the effect of heat shock on LERs in *O. sativa* and *O. australiensis* over a 17-h period (Fig. 2A). There was a significant interaction between species and time of day (*p* < 0.01), indicating that the two species responded to heat differentially throughout the day. For example, LER at 11:00 was ~ 3.17 mm h^−1^ (*O. sativa*) and ~ 3.76 mm h^−1^ (*O. australiensis*); not significantly different. However, after 8 h of heat treatment (15:00), there was a significant ~ 28% reduction in LER in *O. sativa*, while a non-significant increase was registered in *O. australiensis* (~ 4% increase). LERs of the two species remained significantly different from this point until the end of the photoperiod.

After long-term exposure to 35 and 45 °C, LERs in *O. australiensis* but not *O. sativa* increased (*p* < 0.01; Fig. 2B). This interaction was driven by a significant increase in LER for *O. australiensis* when grown at 35 or 45 °C compared with 25 °C (*p* < 0.01) but this was not seen for *O. sativa*.

### Soluble sugars

**Fig. 3:**
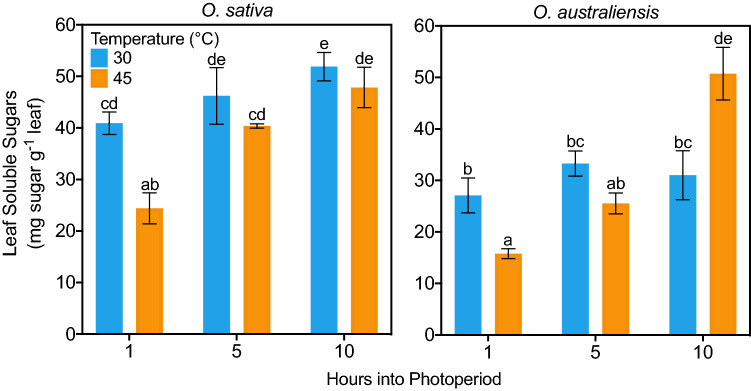
The effect of high temperature (shock) on soluble sugar production in the leaves of *O. sativa* and *O. australiensis* plants 1 h (9:00), 5 h (13:00) and 10 h (18:00) into the photoperiod (see Fig. 1). Plants acclimated to 30 °C were exposed to 45 °C for the entire photoperiod to induce a heat shock. Values are means ± SE of three pot replicates (different to LVDT replicates). Means that do not share a letter are significantly different (Duncan’s Multiple Range test)

A significant three-way interaction showed that the effect of temperature on leaf sugar levels was different for the two species depending on the time of day (Fig. 3; *p* = 0.036). Follow up two-way ANOVAs showed that the interaction between species and temperature (*p* = 0.03) changed throughout the day. That is to say, the effect of temperature on sugar accumulation was different for each species, and this relationship changed throughout the day. This reflects the large increase in soluble sugar concentration in *O. australiensis* leaves at 45 °C 10 h into the photoperiod (*p* = 0.02), indicating an accumulation of sugar in spite of the high temperature. In contrast, the effect of heat on leaf sugar levels was the same for both species 1 h and 5 h into the photoperiod. After 10 h at 45 °C the concentration of sugars in *O. australiensis* leaves equalled that of *O. sativa* leaves.

### Effect of CO_2_ fertilisation on growth and LER

**Fig. 4:**
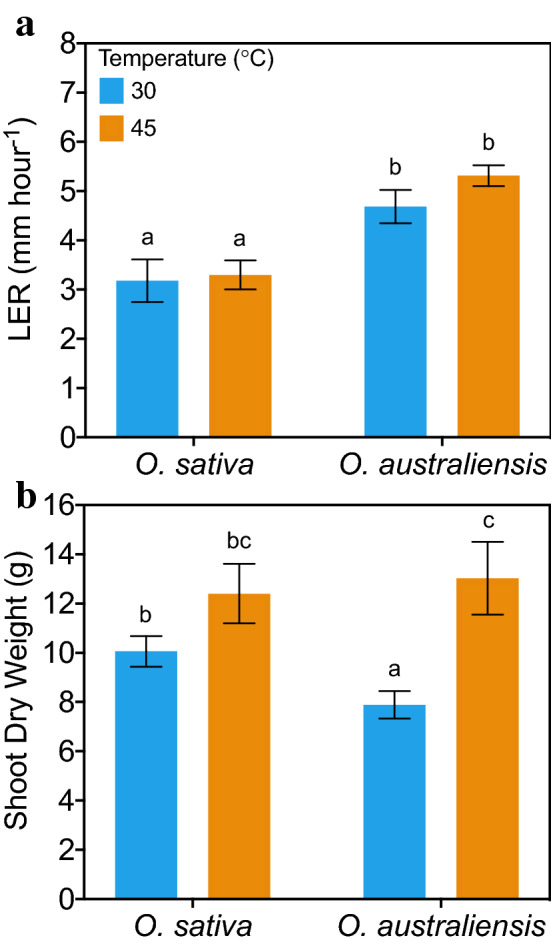
Leaf elongation rates (LERs) and shoot dry weights of *O. sativa* and *O. australiensis* plants grown at 30 and 45 °C under elevated (700 ppm) atmospheric CO_2_ concentrations. **A** LERs are the average elongation rates over 4 h of exposure. This experiment was repeated three times for each group. Values are means ± SE; *n* = 12. **B** Plants (6 weeks old at commencement of experiment) were grown for a further 6 weeks at the indicated conditions before destructive harvesting. Values are means ± SE; n_30C_ = 16, n_45C_ = 4. Means that do not share a letter are significantly different (Duncan’s Multiple Range test)

Contrary to the significantly faster LER of *O. australiensis* leaves when grown at 45 °C in ambient (400 ppm) CO_2_ conditions (Fig. 2B), when CO_2_ levels were raised to 700 ppm the LER of *O. australiensis* was identical at 30 and 45 °C (Fig. 4A). The LER of *O. sativa* was consistently ~ 3 mm h^−1^ at 400 ppm (Fig. 2B) and 700 ppm CO_2_ (Fig. 4A) and was unaffected by atmospheric CO_2_ level. Despite 700 ppm CO_2_ not further accelerating the rapid leaf elongation rate when *O. australiensis* grew at 45 °C, shoot dry mass increased significantly at 45 °C in an elevated CO_2_ atmosphere (*p* < 0.01; Fig. 4B), as was observed for biomass increments at 400 ppm (Fig. 1). On the contrary, shoot dry mass was unaffected by heat when *O. sativa* grew at 700 ppm CO_2_ (Fig. 4B), while growth was slower at 45 °C in 400 ppm CO_2_ (Fig. 1). That is, a 700 ppm CO_2_ atmosphere reversed the impaired growth rates seen in *O. sativa* at 45 °C in 400 ppm CO_2_.

### Net CO_2_ assimilation; *R*_light_, Γ* and ETR

**Fig. 5:**
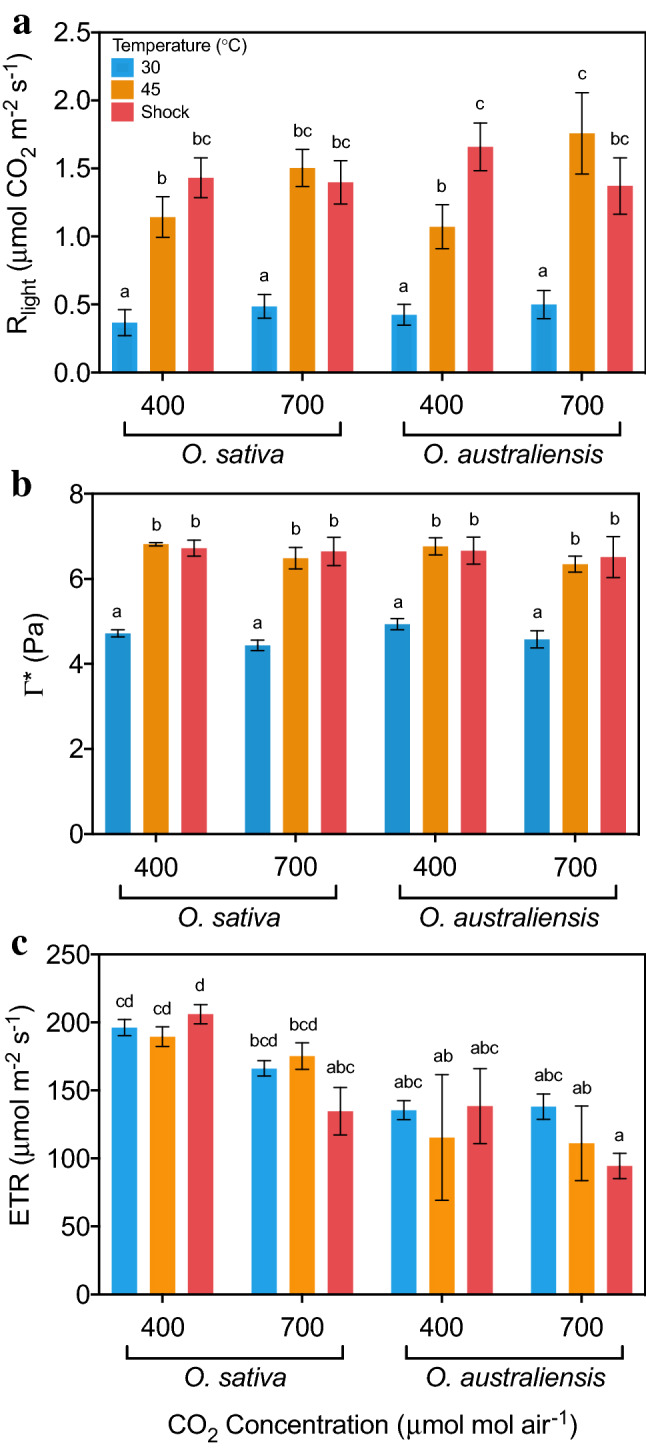
Effect of long- and short-term heat exposure on **A** respiration in the light (*R*_light_), **B** CO_2_ compensation point in the absence of mitochondrial respiration (Γ*), and (C) electron transport rates (*ETR*) in *O. sativa* and *O. australiensis*. Seven-week-old plants were grown at 30 or 45 °C and 400 ppm or 700 ppm for 2 weeks before measurement. The shock group was established by re-measuring the 30 °C plants after one day of exposure to 45 °C. Values are means ± SE (*n* ≥ 5). These measurements were made using a LI-6800. Means that do not share a letter are significantly different (Duncan’s Multiple Range test)

Respiration in the light (*R*_light_) was similar for *O. sativa* and *O. australiensis* (Fig. 5A). Both species had a significantly faster *R*_light_ with rising temperature, and the stimulation of *R*_light_ was not significantly different between the species. The stimulation of *R*_light_ was the same irrespective of a long-term heat exposure or heat-shock, suggesting limited acclimation potential of *R*_light_ for rice or its wild relative. The CO_2_ compensation point of leaves in the absence of respiration (Γ*)—equivalent to the amount of CO_2_ released by leaves via the process of photorespiration—increased significantly with temperature (Fig. 5B). Again, the control 30 °C values and the extent of temperature stimulation was not significantly different between the two species or between the long-term heat exposure and heat shock. Photosynthetic *ETR* determined by chlorophyll fluorescence was significantly different between *O. sativa* and *O. australiensis* irrespective of growth temperature (Fig. 5C), with *O. sativa* having significantly faster *ETR* under all treatments. While *R*_light_ and Γ* did not perform differently with 700 ppm CO_2_ enrichment, the *ETR* of both species declined somewhat with heat shock, though perhaps more so for *O. australiensis*.

### Net CO_2_ assimilation; RACiR curves

**Fig. 6:**
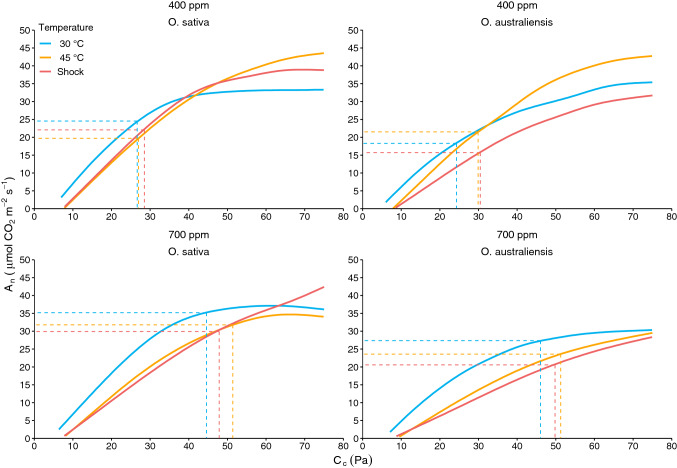
Mean (*n* for each group is reported in Table 1) RACiR curves for *O. sativa* and *O. australiensis* grown under variable temperature and CO_2_ regimes. Seven-week-old plants were grown at 30 or 45 °C and 400 ppm or 700 ppm CO_2_ for 2 weeks in growth cabinets before measurement. The “Shock” group was established by raising the 30 °C growth cabinet temperature to 45 °C for 2 days. *C*_i_ values were converted to *C*_c_ values. Shading around each response curve shows the 95% Confidence Interval for each group. Dotted lines show the mean *C*_c_ values of the plants at their respective growth CO_2_ concentrations and corresponding modelled *A*_n_ rates. Note: inlayed lines do not represent steady-state assimilation rates and thus only provide an indication of CO_2_ assimilation rate response to the specified growth conditions. RACiR curves were generated using a LI-6800

To further characterise the response of photosynthesis in *O. sativa* and *O. australiensis* to temperature, RACiR curves were made and analysed using the standard C_3_ photosynthesis model (Fig. 6, Table 1). CO_2_ carboxylation was the prevailing limitation on *A*_n_ at 400 ppm for both species. Consistent with growth and leaf sugar accumulation, at an ambient CO_2_ of 400 ppm *O. australiensis* had faster and *O. sativa* slower *A*_n_ after acclimation to 45 °C, relative to 30 °C (Fig. 6). The beneficial response of *A*_n_ at 45 °C relative to 30 °C for *O. australiensis* at 400 ppm (Fig. 6) can in part be attributed to overcoming the inherently lower *g*_s_, *C*_i_ and subsequent *C*_c_ concentrations in *O. australiensis* at 30 °C (Table 1). Interestingly, comparisons of long-term 45 °C heat exposure versus heat-shocked plants revealed reduced photosynthetic capacity of *O. australiensis* in heat-shocked plants only, while similar reductions in capacity were observed in *O. sativa* irrespective of exposure time (Table 1). This may suggest potential for acclimation of CO_2_ assimilation to heat in the wild rice but not in *O. sativa*.

**Table 1:** Photosynthetic characteristics of wild and domestic rice grown under variable temperature and CO_2_ regimes, derived from RACiR curves

Temperature (°C)	30				45				45 (Shock)			
[CO_2_] (ppm)	400		700		400		700		400		700	
Species	*O. sat*	*O. aus*	*O. sat*	*O. aus*	*O. sat*	*O. aus*	*O. sat*	*O. aus*	*O. sat*	*O. aus*	*O. sat*	*O. aus*
*V*_Cmax_ (μmol CO_2_ m^−2^ s^−1^)	99.4 ± 7.1 a b	90.2 ± 3.4 ab	102.1 ± 11.4 ab	78.6 ± 1 a	185.2 ± 10.1 c	172.4 ± 18.4 c	167.3 ± 16.5 c	125.2 ± 8.4 b	180.6 ± 14 c	125.3 ± 15.4 b	167.6 ± 6.3 c	111.6 ± 16 a b
*A*_n_/*V*_Cmax_ (%)	24.5	20.2	34.5	34.8	10.6	12.5	19	18.8	12.2	12.5	17.8	18.4
*J*_max_ (μmol m^−2^ s^−1^)	179.2 ± 11 abcde	164.1 ± 5.8 abc	186.2 ± 19.3 abcdef	145.7 ± 3.0 ab	226 ± 6.9 ef	215.3 ± 22.6 def	194.2 ± 16.9 bcdef	145.2 ± 11.3 ab	217.8 ± 16.9 f	165 ± 16.8 abcd	204.7 ± 17.8 cdef	132 ± 16.6 a
*C*_c_ (Pa)	26.6 ± 0.9 ab	24.3 ± 0.5 a	44.6 ± 2.7 c	46.1 ± 2 cd	27.0 ± 0.8 ab	29.9 ± 0.3 b	51.4 ± 1.3 e	51.3 ± 1.6 e	28.5 ± 0.2 ab	30.5 ± 0.6 b	47.9 ± 1.6 cde	49.8 ± 2.8 de
*g*_m_ (μmol CO_2_ m^−2^ s^−1^ Pa^−1^)	10.9 ± 0.1 bcd	5.5 ± 0.07 a	11.2 ± 0.11 bcd	5.4 ± 0.06 a	22.1 ± 0.46 f	10.5 ± 0.26 bc	21.6 ± 0.12 f	11.3 ± 0.32 cd	20.6 ± 0.41 e	10.2 ± 0.26 b	22.4 ± 0.58 f	11.7 ± 0.49 d
*C*_i_ (Pa)	28.9 ± 0.8 a	27.9 ± 0.7 a	47.6 ± 2.5 b	51.1 ± 2 bc	27.9 ± 0.9 a	31.9 ± 0.4 a	52.9 ± 1.2 c	53.4 ± 1.7 c	29.6 ± 0.2 a	32 ± 0.6 a	49.2 ± 1.7 bc	51.6 ± 2.9 bc
Leaf Temperature (^o^C)	28.8 ± 0.2 a	29.5 ± 0.2 a	29.6 ± 0.2 a	29.6 ± 0.2 a	41.5 ± 0.4 d	38.9 ± 1.1 bc	41.1 ± 0.1 cd	41.6 ± 0.5 d	40.2 ± 0.4 bc	39.4 ± 0.9 b	41.7 ± 0.5 d	42.2 ± 0.7 d
*g*_s_ (mol H_2_O m^−2^ s^−1^)	0.64 ± 0.08 abc	0.49 ± 0.1 abc	0.34 ± 0.04 a	0.4 ± 0.05 ab	0.67 ± 0.08 bc	0.77 ± 0.08 cd	0.6 ± 0.003 abc	0.53 ± 0.11 abc	0.84 ± 0.15 d	0.79 ± 0.09 cd	0.45 ± 0.08 ab	0.39 ± 0.11 ab
*n*	5	6	5	4	6	5	6	6	6	5	5	4

The shock group was made up of 30 °C plants exposed to 45 °C for 2 days. All values are means (± SE), except for *A*_n_/*V*_cmax_ (%). Means that do not share a letter are significantly different (as determined by Duncan’s Multiple Range Test)

*A*_n_ CO_2_ assimilation rate measured on LI-6800, *V*_Cmax_ maximum velocity of Rubisco, *J*_max_ maximum velocity of electron transport, *C*_C_ pressure of CO_2_ at the chloroplast, *g*_m_ mesophyll conductance, *C*_i_ pressure of intercellular CO_2_, *g*_s_ stomatal conductance to water, *n* number of replicate RACiR curves per group

In plants acclimated to a [CO_2_] of 700 ppm, overall photosynthetic capacity was significantly reduced for *O. australiensis* at 45 °C. This was demonstrated by a lower *A*_n_ and substantially reduced *V*_cmax_ and *J*_max_ at 45 °C and 700 ppm CO_2_ relative to 400 ppm (Fig. 6; Table 1). In contrast, *O. sativa* had an increase in *A*_n_ and a more limited suppression of *V*_cmax_ and *J*_max_ when grown at 45 °C and exposed to 700 ppm. For example, at 45 °C and 700 ppm the *V*_cmax_ of *O. australiensis* was 66% of the rate at 400 ppm, while for *O. sativa*, *V*_cmax_ remained at 93% of the rate at 400 ppm. A greater downregulation of photosynthetic capacity under heat and high CO_2_ was therefore seen in *O. australiensis* compared with *O. sativa*. Significant suppression of photosynthetic capacity of *O. australiensis* exposed to high CO_2_ and heat was accompanied by greater photosynthetic efficiency under these conditions, and therefore little change in *A*_n_ was observed (Table 1).

### Relative abundance of Rca as a percentage of Rubisco

**Fig. 7:**
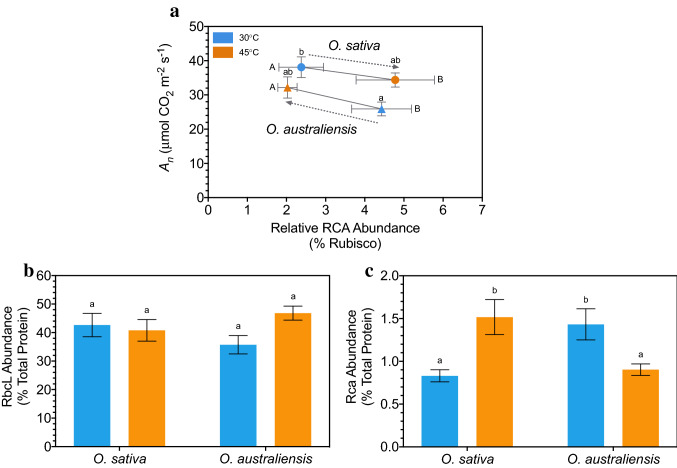
Effect of heat on CO_2_ assimilation and the abundance of RbcL and Rca in *O. sativa* and *O. australiensis.* **A** Relationship between the abundance of Rca relative to Rubisco (Rca emPAI/RbcL emPAI) and net CO_2_ assimilation (*A*_n_) in *O. sativa* and *O. australiensis* leaves exposed to 30 and 45 °C (shock). Arrowheads emphasise the relationship between relative Rca abundance and photosynthetic rates for each species. *A*_n_ was estimated using a LI-6400. Capital letters show differences in Rca relative abundance; lowercase letters show differences in assimilation rates. **B** Abundance of RbcL relative to total protein emPAI (RbcL emPAI/total emPAI). **C** Abundance of Rca relative to total protein emPAI (Rca emPAI/total emPAI). Values are means ± SE of nine pot replicates (time of day replicates were pooled). Means that do not share a letter are significantly different (Duncan’s Multiple Range test). See Fig. 2 for sampling information

The effect of temperature on the relative abundance of Rca as a proportion of Rubisco for *O. sativa* and *O. australiensis* was determined (Fig. 7). At 45 °C the abundance of Rca as a proportion of RbcL was significantly reduced in *O. australiensis*. In spite of this, *A*_*n*_ increased in *O. australiensis*. The opposite was observed for *O. sativa*—at 45 °C the abundance of Rca as a proportion of RbcL was significantly increased, while photosynthetic rates declined.

There was a significant interaction between species and temperature (*p* < 0.001). At 30 °C, the concentration of Rca relative to Rubisco in *O. australiensis* leaves was ~ 2.2 times greater than it was at 45 °C. Conversely, in *O. sativa* leaves the concentration of Rca relative to Rubisco at 30 °C was half that at 45 °C (Fig. 7a). Differences in Rca abundance relative to RbcL were not strictly due to differences in the abundance of RbcL but were driven more by changes in the abundance of Rca (Fig. 7b, c). Despite the relative abundance of Rca decreasing at 45 °C in *O. australiensis*, steady-state *A*_n_ appeared to increase (measured on the same leaves as those sampled for protein; Fig. 7a). By contrast, the increase in relative Rca abundance in *O. sativa* was associated with decreased *A*_n_ with heat.

A two-way interaction between temperature and time of day (*p* < 0.01) shows that the abundance of Rca relative to RbcL increased following exposure to 45 °C for 5 h, though no other significant differences were detected (Fig. S2; Tukey HSD). Similarly, a two-way interaction between species and time of day (*p* < 0.001) shows that Rca:RbcL increased 5 h into the photoperiod in *O. sativa*, and this ratio was greater than that seen in *O. australiensis* under the same conditions (Tukey HSD).

## Discussion

This study explores two closely related grasses in which we have previously established starkly contrasting degrees of thermotolerance (Scafaro et al. 2011, 2016). The tolerant species, *Oryza australiensis*, is an extremophile distributed across the hot northern savannah of Australia, while *O. sativa* ssp. *japonica* is largely cultivated in the humid sub-tropics where daily maxima rarely exceed 35 °C. However, the mechanisms that enable wild rice to continue growing, even during sustained periods of high temperature, are still not known. This paper links photosynthetic processes in *O. australiensis* with its sustained thermotolerance during early vegetative growth.

When grown at high temperature (45 °C) and ambient CO_2_ (400 ppm), *O. australiensis* accumulated more biomass than *O. sativa*. Furthermore, *O. australiensis* was susceptible to low temperature (25 °C) while *O. sativa* suffered severe growth penalties when grown at high temperature. Ali et al. (2019) showed reductions in growth of the same *O. sativa* cultivar (Amaroo) when grown at high temperature for a short time during grain filling. Furthermore, Perdomo et al. (2015) show reduced biomass accumulation in *O. sativa* (cv. Bomba) when grown at high temperature. Taken together these results underline the superior thermotolerance of *O. australiensis* to sustained heat, relative to *O. sativa*. Interestingly, 45 °C days only slowed biomass gain in *O. sativa* after 3 weeks, even though leaves elongated more slowly within a few hours of 45 °C being imposed. Previous studies have highlighted the impact of heat stress on the photosynthetic and respiratory rates and subsequent biomass accumulation of *O. sativa* (Jagadish et al. 2015; Shi et al. 2016; Zhang et al. 2018; Karwa et al. 2020). While these studies only consider the impact of heat over a relatively short time (max. 20 days), and usually during or after anthesis with a focus on grain yield, they reveal alterations to rice carbon metabolism initiated by high temperatures. In this context, the results of the present study indicate that vegetative growth in *O. sativa* is impeded at sustained high temperature by its cumulative impact on the whole-plant carbon budget. We ascribe this to reduced carbohydrate availability and mobilisation from reserves rather than a shift in partitioning of biomass between roots and shoots because root-to-shoot ratios were relatively stable over time (Fig. S3).

LER data showed that leaves of both species elongated rapidly 1–2 h after transfer to 45 °C, which suggests that heat did not directly impair the mechanics of cell expansion, for example by disrupting the cytoskeleton (Smertenko et al. 1997) or membrane integrity (Horváth et al. 2012) but rather it acted through secondary impacts of heat on metabolism. The species contrast was first seen 4 h after plants were exposed to 45 °C, consistent with a growing deficit of carbohydrates in *O. sativa* and subsequent slowing of leaf growth over the diurnal cycle of heat. This is in accord with the finding that the impact of heat on grain filling in *O. sativa* becomes more severe with prolonged exposure to heat (Shi et al. 2016). On the other hand, LER in *O. australiensis* was essentially maintained above 3 mm h^−1^ for 10 h at 45 °C, while rates returned to 1—1.5 mm h^−1^ in both species at night. These findings corroborate those of Scafaro et al. (2010) and Scafaro et al. (2016), who showed reductions in LER of *O. sativa* following 4 h of exposure to 45 °C of up to 50%, while Scafaro et al. (2016) report no significant reduction in the LER of *O. australiensis* after 4 h at 45 °C, which was linked to its photosynthetic thermotolerance. That *O. australiensis* has consistently demonstrated superior growth to *O. sativa* in the face of high temperature suggests that it has access to the resources (namely carbon resources either generated via photosynthesis or mobilised from stored carbohydrates) that are required to support such growth.

We found that after 10 h at 45 °C, sugar concentrations accumulated threefold in *O. australiensis* and doubled in *O. sativa* (Fig. 3). These data contrast with the steady sugar levels recorded in rice leaves exposed to longer periods at less extreme temperatures up to 40 °C (Rashid et al. 2020). Soluble sugars are thought to play a role in various abiotic stress tolerances by acting as signalling molecules, by conferring antioxidant properties (Harsh et al. 2016), by acting as compatible solutes (Kaplan and Guy 2004) and stabilising protein structure (Lee and Timasheff 1981). Given the role that soluble sugars play in protecting plants against thermally induced damage, we speculate that high soluble sugar levels in both rice species contributed to thermotolerance at the molecular level. In addition, the species-specific accumulation of sugars in response to heat is interesting as a resource for growth and photosynthetic rates. Steady-state soluble sugar concentrations in leaves are the consequence of assimilation rates, starch degradation, and sugar utilisation (Stitt and Zeeman 2012). Therefore, trebling of leaf soluble sugars in *O. australiensis* should be seen in the context of the faster growth at 45 °C compared with *O. sativa*; leaf growth in *O. australiensis* continued unabated at 45 °C while *O. sativa* leaves elongated significantly slower (Fig. 2A, B). This makes the marked accumulation of soluble sugars in leaves of *O. australiensis* remarkable because it occurred despite a greater demand for carbohydrates that would be required to sustain steady growth. The maintenance of *O. sativa* leaf sugar levels at 45 °C may have come at a cost to starch reserves, considering photosynthesis was impaired under these hotter growth conditions in the domesticated rice (Figs. 6, 7). Increasing soluble sugar content in plant tissues is often reported alongside reductions in starch reserves as stored carbohydrates are mobilised, requiring the expression of a suite of genes involved in starch degradation and monosaccharide transport (Kaplan et al. 2006). For example, Yichie et al. (2019) reported increases in expression of a starch synthase in a salt-susceptible population of wild rice, while a tolerant population of the same species had increased expression of monosaccharide transporters. The rapid slowing of biomass accumulation in *O. sativa* after 28 days at 45 °C (Fig. 1) may in fact be due to starch reserves reaching a critically low level after sustained depletion.

Contrasting sugar levels in the two species and previous observations that photosynthesis is vulnerable to heat-induced damage at 45 °C (Salvucci et al. 2001; Sage et al. 2008; Scafaro et al. 2010, 2016; Yamori et al. 2014; Busch and Sage 2017) led to an inquiry into photosynthetic metabolism in the two species under heat shock. Unsurprisingly, peak *A*_*n*_ recorded (as instantaneous measurements) at 30 °C were 50% higher in the domesticated cultivar of *O. sativa*, while at 45 °C rates in *O. sativa* and *O. australiensis* were approximately equal (Fig. 7). This finding emphasises the highly significant opposing effects of heat on assimilation in the two species (i.e. a 30% increase in *O. australiensis* and a 20% decrease in *O. sativa* when exposed to 45 °C). Similarly, IR64 (an *O. sativa* cultivar) had reduced photosynthesis when exposed to a high temperature shock (Rashid et al. 2020). Furthermore, *O. sativa* (cv. Bomba) had reduced daily average assimilation rates when grown at high temperature (Perdomo et al. 2015). Importantly, the changes in instantaneous measures of *A*_*n*_ during heat shock could not be explained by differences in stomatal conductance (*g*_s_), intracellular CO_2_ (*C*_i_), leaf temperature nor vapour pressure deficit (Fig. S4). This finding is supported by a preliminary study we performed, where significant changes in VPD could not account for differences in assimilation rates because, despite the response of *g*_s_ to high temperature, intercellular CO_2_ availability was the same for both species (Fig. S5). Differences in leaf anatomical properties between rice and its wild relatives do exist, and these differences seem to influence CO_2_ conductance properties between the air and chloroplasts (Scafaro et al. 2011; Giuliani et al. 2013). However, the results presented here suggest that temperature-dependent changes in *A*_*n*_ are dictated by changes in the efficiency of the underlying photosynthetic biochemistry.

RACiR curves are a novel technique that have been used successfully to rapidly acquire gas exchange data for the estimation of *V*_cmax_ and *J*_max_ while simultaneously overcoming the issues of stomatal attenuation and changes in enzyme kinetics that are characteristic of the traditional *A*:*C*_i_ curve (Stinziano et al. 2017, 2019; Coursolle et al. 2019; Vincent et al. 2020). RACiR curves, Laisk curves, and point measures of chlorophyll fluorescence showed that differences in assimilation between wild and domestic rice during long- or short-term heat exposure could not be explained by differences in photorespiration, respiration or *ETR* (Fig. 5). That is, the thermotolerance of assimilation in *O. australiensis* cannot be due to a species-specific response of photorespiration, respiration, or *ETR* to heat.

Rates of photorespiration increase with increasing temperature because the specificity of Rubisco to CO_2_ declines relative to O_2_ under heat, and the abundance of CO_2_ relative to O_2_ is reduced due to differences in the solubility of these two gases at high temperature (Walker et al. 2016; Dusenge et al. 2019). Given that rates of photorespiration are driven in part by the affinity of Rubisco for CO_2_, we conclude that the lack of species differences observed here indicate that the wild and domestic rice Rubisco isoforms must have similar affinities for their substrates. This is in accordance with recent work on *Arabidopsis* that showed no genetic basis for variation in photorespiration between ecotypes (Tomeo and Rosenthal 2018).

Similarly, respiration increases with increasing temperature (Dusenge et al. 2019). Here, we showed similar increases in respiration at high temperature in both species. Rashid et al. (2020) showed that rice (IR64) had limited respiratory acclimation over seven days of exposure to 40 °C, mirroring the results obtained here. Interestingly, *O. australiensis* respiration showed greater potential to acclimate to sustained high temperature at 400 ppm CO_2_ than *O. sativa*. Thus, while we cannot attribute differences in assimilation to the observed differences in respiration between the species at high temperature, *O. australiensis* may provide useful germplasm to explore variation in respiratory response under heat. Finally, reduced *A*_n_ in *O*. *sativa* at 45 °C (both long- and short-term) comes about even though concentrations of CO_2_ at the chloroplast remained stable or increase. Taken together, the results presented thus far provide evidence for the notion of biochemical limitations at the chloroplast during episodes of high temperature in *O. sativa* but not *O. australiensis*. Interestingly, the RACiR curves showed some degree of acclimation of assimilation to both long-term heat (*cf*. short-term) and elevated ambient CO_2_ in *O. australiensis* though not in *O. sativa* (Fig. 6). The downregulation of photosynthetic capacity in *O. australiensis* when grown at 700 ppm CO_2_ suggests that the wild species is capable of meeting sink demands even when reducing its investment in photosynthetic machinery. This is seen in its most extreme form in *O. australiensis* plants grown at 700 ppm CO_2_ *and* sustained high temperature, which may indicate a metabolism that is adapted to operate most efficiently in hot environments. On the other hand, *O. sativa* appears to maintain investment in photosynthetic machinery at high CO_2_, resulting in much faster *A*_n_ with CO_2_ enrichment. Under a similar 700 ppm CO_2_ enrichment study, *O. sativa* significantly increased *A*_n_ while the wild relative *Oryza meridionalis* had a limited response (Rahman et al. 2018). The *A*_n_ of wild relatives thus seems to be less responsive to CO_2_ fertilisation relative to domestic rice. We speculate that *O. australiensis* reduced photosynthetic capacity with CO_2_ enrichment because of limited sink demand, whereas *O. sativa* maintained photosynthetic capacity due to artificial selection increasing demand for assimilates needed for grain filling. Supporting this postulate, enriched CO_2_ has consistently been shown to increase grain yield in commercial rice (Madan et al. 2012), demonstrating that improved crop performance by engineering faster assimilation will likely be effective only in cases where demand for assimilates (sink strength) has not already reached saturation.

The CO_2_ response curves suggested Rubisco carboxylation limitations existed in *both species* at 45 °C and 400 ppm CO_2_, and thus a direct effect of heat on CO_2_ fixation by Rubisco is strongly implicated as the reason for differences in photosynthetic thermotolerance between the species. Prior evidence (Scafaro et al. 2016) suggests that the properties of Rca form part of the explanation for heat tolerance in *O. australiensis*. Indeed, the photosynthetic heat tolerance of *O. sativa* can be improved with overexpression of *O. australiensis* or maize Rca (Yamori et al. 2012; Scafaro et al. 2018). The activation of Rubisco by Rca depends on the ratio of the two enzymes and the susceptibility of Rca to heat (Perdomo et al. 2017). Rca abundance can respond dynamically to heat stress in a timeframe of hours to days through transcriptional and post-transcriptional regulation (Degen et al. 2021). In relation to the ratio of Rubisco to Rca, Fukayama et al. (2012) and Fukayama et al. (2018) showed that rice overexpressing functional Rca had reduced abundance of Rubisco, while an Rca knock-out line had increased Rubisco abundance. Suganami et al. (2018) showed that as the abundance of Rubisco increases, cells disinvest in Rca, and vice versa, indicating that this feedback operates in both directions. Further, a recent report in rice demonstrated that overexpression of both Rca and Rubisco leads to improvement in assimilation, particularly under heat stress (Qu et al. 2021). Given that Rca functions in the activation of Rubisco (Portis 2003), these previous results imply that photosynthetic capacity is tightly regulated and set by the number of active Rubisco sites. As we observed changes in Rca abundance, but no significant changes in Rubisco abundance with heat, Rca was regulating photosynthetic capacity. Contrary changes in total Rca abundance between the species when grown at 45 °C complement the inherent thermostability of the Rca isoforms in each species. Specifically, the dominant Rca β isoform found in *O. australiensis* is known to have greater thermostability than its *O. sativa* ortholog (Scafaro et al. 2016). Thus, it may be the case here that at 30 °C *O. australiensis* Rca was operating below its thermal optimum. An increase in abundance of Rca presumably compensated for the loss in efficiency at this sub-optimal temperature (Fig. 7). This limitation was presumably lifted at higher temperatures, allowing for increased enzyme function and a decrease in Rca abundance. Interestingly, reduced investment in Rca because of increased Rca activity may enhance plant growth over long periods of heat because after Rubisco, Rca is the second highest consumer of ATP in leaves (Li et al. 2017). Furthermore, the savings in ATP costs associated with reduced Rca may contribute to the greater respiratory acclimation potential of hot grown *O. australiensis,* as we explore above. We suggest that the opposite applies in *O. sativa*, whose Rca may have been operating outside its thermal optimum at 45 °C, likewise explaining the increased accumulation of the enzyme during exposure to heat and reduced accumulation under optimal conditions (30 °C). When grown at high temperatures, the stability of Rca may therefore explain the reduced Rca abundance, greater photosynthetic performance, and ultimately faster growth in the heat-adapted *O. australiensis*. This is supported by our earlier evidence that transgenic rice expressing the thermotolerant ortholog of Rca from *O. australiensis* had increased growth rates and seed yield at 45 °C (Scafaro et al. 2018). We further demonstrate in this study that neither photosynthetic electron transport, respiratory capacity nor photorespiratory performance could explain the susceptibility of *O. sativa* to extreme heat. Furthermore, we found no evidence for differences in Rubisco’s affinity for CO_2_ in the wild rice, which would have provided partial explanation for improved assimilation at high temperature. Rca as a driver of heat tolerance in wild rice therefore seems highly likely (Perdomo et al. 2017). Whether this mechanistic basis of heat tolerance applies to other important crops should be a priority of future research.

## Supplementary Information

Below is the link to the electronic supplementary material.

Supplementary file1 (DOCX 362 KB)

## Data Availability

All data available upon request to the corresponding author.

## Code availability

All code and data available at: https://github.com/aaronphillips7493/Oa_heat_paper_2021.
