# Supplementary material for: Photosynthetic traits of Australian wild rice (Oryza australiensis) confer tolerance to extreme daytime temperatures
Source: Plant Mol Biol. 2022 Jan 8;110(4-5):347–63. doi: 10.1007/s11103-021-01210-3 (PMC9646608; doi:10.1007/s11103-021-01210-3)
Supplement: Supplementary file 1 — Supplementary file1 (DOCX 362 KB) [file 11103_2021_1210_MOESM1_ESM.docx]

**Supplementary Information**

**Title:**

Photosynthetic traits of Australian wild rice (*Oryza australiensis*) confer tolerance to extreme daytime temperatures

**Journal:**

Plant Molecular Biology

**Authors:**

Aaron L. Phillips^1, 3, 4^, Andrew P. Scafaro^2^, Brian J. Atwell^3*^

**Author Affiliations:**

^1^University of Adelaide, School of Agriculture, Food, and Wine, Adelaide, South Australia, Australia

^2^The Australian National University, ARC Centre of Excellence in Plant Energy Biology, Research School of Biology, Canberra, Australian Capital Territory, Australia

^3^Macquarie University, Department of Biological Sciences, Sydney, NSW, Australia

^4^ARC Centre of Excellence in Plant Energy Biology, School of Agriculture, Food, and Wine, The University of Adelaide, Adelaide, SA, 5064, Australia

***Corresponding Author**:

Brian Atwell

brian.atwell@mq.edu.au

+61 (2) 9850 8224

**Fig. S1**: Root dry weight of *O. australiensis* and *O. sativa* grown at 25, 35 and 45°C.

**Fig. S2**: Rca:RbcL in temperature-treated *O. australiensis* and *O. sativa* leaves throughout the photoperiod.

**Fig. S3**: Root to shoot ratio (dry weights) of *O. australiensis* and *O. sativa* grown at 25, 35 and 45°C.

**Fig. S4**: (a) CO2 assimilation rates, (b) Stomatal conductance, (c) intercellular leaf CO_2_ concentration, (d) leaf temperature, and (e) vapour pressure deficit of *O. australiensis* and *O. sativa* exposed to 30°C (blue line) or 45°C (orange line).

**Fig S5.** CO_2_ assimilation, vapour pressure deficit, stomatal conductance, and intercellular CO_2_ concentration of *O. australiensis* and *O. sativa* plants grown at 30°C or 45°C and 400 or 700 ppm CO_2_ (n = 6).
